# Falling rates but projected rising numbers of fractures in elderly Norwegians: a study of fracture rates in the Norwegian patient registry from 2010 to 2021, extrapolated to 2041

**DOI:** 10.2340/17453674.2024.42634

**Published:** 2025-02-24

**Authors:** Jørgen ANDVIG, Lars G JOHNSEN, Sara M NILSEN, Gudrun W BJØRNELV, Andreas ASHEIM

**Affiliations:** 1 Department of Orthopaedic Surgery, Molde Hospital, Klinikk SNR, Møre & Romsdal Health Trust; 2 Department of Public Health and Nursing, Norwegian University of Science and Technology; 3 Department of Orthopaedic Surgery, St. Olavs Hospital, Trondheim University Hospital, Trondheim; 4 Department of Neuromedicine and Movement Science, Faculty of Medicine and Health Sciences, NTNU-Norwegian University of Science and Technology, Trondheim; 5 Center for Health Care Improvement, St. Olav’s University Hospital, Trondheim; 6 Department of Clinical and Molecular Medicine, Faculty of Medicine and Health Sciences, Norwegian University of Science and Technology, Trondheim; 7 Department of Health Management and Health Economics, University of Oslo; 8 Norwegian University of Science and Technology, Department of Mathematical Sciences, Trondheim, Norway

## Abstract

**Purpose:**

Our aim was to calculate rates of major fractures by fracture location in elderly Norwegians over the years 2010 to 2021 and thereby estimate the volume of fractures in this population by 2041.

**Methods:**

We identified fractures in persons aged 65 years and over from the Norwegian Patient Registry. We summarized age- and sex-specific numbers of fractures and incidence rates by fracture location. Extrapolating adjusted incidence rates combined with population projections from Statistics Norway, we estimated the expected numbers of fracture cases for the following 20 years.

**Results:**

The total number of major fractures rose from 22,581 in 2010 to 27,596 in 2021. While the number of hip fractures was relatively stable (8,164 to 8,194 over the period), there were substantial increases in the number of fractures in the upper extremities, spine and pelvis, and lower extremities. Annual changes in incidence rates adjusted for age and sex were 0.6% (95% confidence interval [CI] 0.4–0.7), 1.2% (CI 0.9–1.4), 0.4% (CI 0.1–0.7), and –1.9% (CI –2.0 to –1.7) for upper extremity, spine and pelvis, lower extremity, and hip respectively. Extrapolating trends in incidence rates, we estimate a 64% (95% prediction interval 48–70) overall increase in the number of major fractures by 2041 compared with 2021, primarily due to the aging of the population.

**Conclusion:**

Incidence rates of hip fractures decreased over the period, while rates of other major fractures increased. We can expect a substantial increase in the number of fractures over the coming years, primarily due to the expected aging of the population.

With growing elderly populations in industrialized countries, an increase in fragility-related fractures can be expected, leading to higher healthcare demands and costs for individual patients and society [1]. While previous studies have sought to quantify the growing burden of fractures in the elderly, hip fractures have received the most attention due to a high relative incidence, morbidity, and cost of care. Rates of other fractures in the elderly are less well documented despite carrying significant morbidity and costs [2,3]. Using comprehensive data to quantify fracture rates and the expected changes for the coming years may serve as the basis for policies on resource allocation for prevention and treatment strategies.

Few nationwide studies measuring overall fracture rates exist. Some attempts have been made to estimate total fracture numbers by imputation from hip fracture rates [1,4]. It is not evident whether this correlates precisely to actual rates of other fractures. Norway benefits from a universal access public healthcare system, and nearly all fracture cases receive medical attention in a public hospital. All hospital contacts are recorded in national registries coupled to unique personal identification numbers. This allows for consistent, nationwide data collection and analyses over time.

The aim of this study was to describe change in annual incidences rates of common major fractures in the elderly population presenting to Norwegian hospitals from 2010 to 2021 and estimate the expected rates of fractures over the next 20 years.

## Methods

We followed the STROBE guidelines for reporting on cohort studies.

### Setting

The Norwegian healthcare system has a universal access, single-payer structure that serves the entire national population. Specialist care is provided at geographically dispersed clinics and hospitals [5]. Fracture care is almost exclusively managed in public hospitals.

### Data

Norwegian residents all have unique personal identification numbers, allowing linkage of patient-level data to national registries, including healthcare services from the Norwegian Patient Registry (NPR) and demographic and socioeconomic data from Statistics Norway [6].

All treatments provided by publicly funded healthcare services are reported to the NPR [6]. Diagnoses are coded according to the International Statistical Classification of Diseases and Related Health Problems (ICD-10) [7]. Fracture diagnoses in the reporting system have been found to have high positive predictive values when also employing wash-out to exclude follow-up [8].

From Statistics Norway we had access to data on year of birth, date of death, sex, municipality of residence, immigration, and emigration status. These data were linked to the data from the NPR on the individual level using de-identified keys. Statistics Norway also publishes population projections for future demographic development by age and sex in “low,” “main,” and “high” alternatives, according to different projections of fertility, death rates, immigration, and emigration [9]. The main alternative is considered closest to prognostic.

### Study population

We collected data from the NPR linked to data from Statistics Norway, assembling a nationwide study population of individuals 65 years or older from 2010 to 2021, identifying acute hospital episodes with an ICD-10 code corresponding to a primary diagnosis as specified in Supplementary Table S1 [7]. We included fractures of the major bones of the upper extremity including the shoulder girdle, the spine and pelvis, and lower extremity, thus excluding the skull, ribs, and minor bones of the wrist, hand and foot. We grouped the fractures into 4 anatomic groups with ICD-10 codes as follows: Upper extremity including shoulder girdle: S42.0–4, S52.0–6; Spine and pelvis: S12.0–9, S22.0, S22.1, S32.0, S32.1–5, S32.7; Lower extremity excluding hip: S72.3, S72.4, S72.7, S82.0–8; Hip: S72.0–2. We include hip fractures as a separate group for benchmarking purposes, being the single most common and most extensively studied fracture type in this age group.

**Figure 1:**
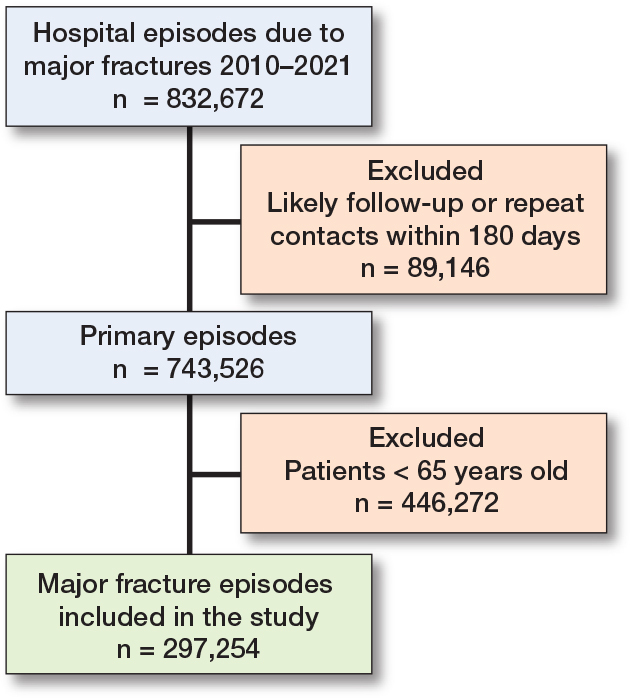
Flowchart of the selection of fracture cases for inclusion from the NPR for the years 2010 to 2021

We applied a series of measures to ensure that each fracture was counted only once. First, we excluded hospital contacts related to follow-up. Second, we imposed a 180-day wash-out period for the same fracture location in the same individual to exclude follow up not coded as such [8]. A flowchart showing the selection of the cohort is shown in Figure 1.

### Statistics

We summarized yearly numbers of fractures and incidence rates in total, by anatomic group, and by fracture location. Person years at risk was calculated as years alive from the year a person turned 65 years old until death or emigration.

We calculated adjusted incidence rates using a Poisson regression model, adjusted for sex, and age with a quadratic term, using an offset parameter to account for time at risk. This model was subsequently used to project the number of fractures per anatomical group up to the year 2041, accounting for anticipated changes in demographic composition. 3 alternatives for future population development were used to encompass the inherent uncertainty in the projections. We used a linear extrapolation of incidence rates, based on the development from 2010 to 2021, presented with bootstrapped 95% prediction intervals (PI). As a sensitivity analysis, we also extrapolated using a constant incidence rate, fixed at the 2021 level. To study incidence rates stratified to patient age we divided the population into 5-year age brackets (65–69, 70–74, 75–79, 80–84, 85–89, 90–94, 95–100, > 100), presenting fracture rates graphically for men and women.

Computations were done in Stata (Version 16, StataCorp LLP, College Station, TX, USA).

### Ethics, registration, data sharing, use of AI, funding, and disclosures

The study received approval from the Regional Committee of Ethics in Medical Research (2016/2159), and participant consent was not required. The study was not pre-registered.

The study data can be accessed from Norwegian registries at helsedata.no. However, availability is restricted. This data was used under license for the current study and is not publicly accessible. AI tools were not used.

JA receives research grants from the Liaison Committee between the Central Norway Regional Health Authority (RHA, Klinikk SNR) and the Norwegian University of Science and Technology (NTNU). AA and SMN were funded by Norwegian Research Council grant number 295989.

The authors declare that they have no conflicts of interests. Complete disclosure of interest forms according to ICMJE are available on the article page, doi: 10.2340/17453674.2024.42634

## Results

We identified 832,672 major fractures from 2010 to 2021. After excluding likely repeat contacts/ follow up (n = 89,146) and persons < 65 years of age (n = 446,272) we included 297,254 fractures for analysis (Figure 1).

These fractures occurred in 232,737 individuals. The overall mean incidence rate was 2,808 fractures per 100,000 person-years. Women made up 74% of cases. Median ages were slightly lower for individuals with fractures of the extremities compared with those in the spine/pelvis and hip fracture groups (Table 1).

**Table 1:** Characteristics of the study population

Factor	All major fractures	Upper extremity	Spine and pelvis	Lower extremity excluding hip	Hip
Fractures (% of total)	297,254	112,977 (38)	39,349 (13)	46,053 (15)	98,875 (33)
Unique patients, n	232,737	99,869	36,937	42,269	91,572
Incidence rate ^a^	2,808	1,067	373	435	938
Women, n (%)	219,699 (74)	90,964 (81)	26,447 (67)	33,605 (73)	68,683 (69)
Men, n (%)	77,555 (26)	22,013 (19)	12,902 (33)	12,448 (27)	30,192 (31)
Age women, median (IQR)	80 (72–87)	76 (70–84)	84 (77–89)	76 (70–84)	85 (77–89)
Age men, median (IQR)	78 (71–85)	74 (68–80)	79 (72–86)	73 (69–82)	83 (72–86)

a per 100,000 person-years

IQR = interquartile range.

### Trends from 2010 to 2021

**Figure 2:**
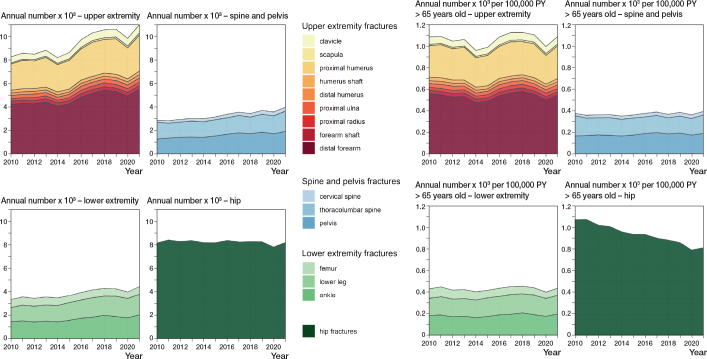
Annual number of major fractures and incidence rates for the years 2010 to 2021. Stacked areas display the sum of individual component fractures in each of the 4 anatomic groups.

The total annual number of major fractures increased from 22,581 in 2010 to 27,596 in 2021. The annual number of fractures in the upper extremities increased from 8,256 to 10,991, spine and pelvis fractures from 2,854 to 3,965, and lower extremity fractures from 3,307 to 4,446 between 2010 and 2021 (Figure 2, Supplementary Tables S2–S4 respectively). Conversely, the number of hip fractures remained relatively stable in the same period (8,164 to 8,194).

Person-years at risk in the study population increased by 33% from 758,000 to 1,006,000 from 2010 to 2021 (Supplementary Table S2). This increase was higher in men (42%) than in women (26%). Overall, incidence rates of major fractures fell slightly from 2,979 per 100,000 person-years in 2010 to 2,743 in 2021. Incidence rates fell more in women (7%) than in men (3%). We find relatively unchanged incidence rates for all the anatomic fracture groups with minor increases, except for hip fractures, which show a marked reduction in incidence (Figure 2).

The estimated crude change in incidence rate for all fractures 2010 to 2012 was –0.8% per year (95% confidence interval [CI] –0.9 to –0.7), driven by a reduction in incidence rate of hip fractures with a –2.7% yearly change (CI –2.9 to –2.5) (Table 2). Conversely, the incidence rates for spine and pelvis fractures increased by 0.5% (CI 0.2–0.8). The other fracture groups had smaller increases in incidence rates (Supplementary Table S5). After adjusting incidence rates for differences in composition of age and sex over time, we estimate increased incidence rates in all anatomic groups except hip fractures, where the decrease became less pronounced (Table 2). For all major fractures combined the result was a near constant adjusted incidence rate, with a yearly change of –0.2% (CI –0.3 to –0.1).

**Table 2:** Annual percentage changes in fracture number, crude incidence rates and incidence rates adjusted for sex and age with a quadratic term

Factor	Annual percentage change (CI)		
	Fracture number	Crude incidence rate	Adjusted incidence rate
All major fractures	1.7 (1.6 to 1.9)	–0.8 (–0.9 to –0.7)	–0.2 (–0.3 to –0.1)
Upper extremity	2.7 (2.6 to 2.9)	0.2 (0.0 to 0.4)	0.6 (0.4 to 0.7)
Spine and pelvis	3.1 (2.8 to 3.4)	0.5 (0.2 to 0.8)	1.2 (0.9 to 1.4)
Lower extremity excluding hip	2.6 (2.3 to 2.9)	0.1 (–0.2 to 0.4)	0.4 (0.1 to 0.7)
Hip	–0.2 (–0.4 to –0.1)	–2.7 (–2.9 to –2.5)	–1.9 (–2.0 to –1.7)

### Projections of future fracture volumes

**Figure 3:**
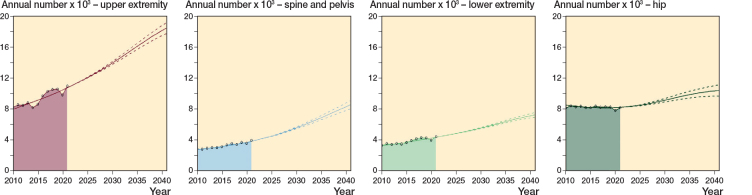
Projected numbers of major fractures in the Norwegian elderly population per year until 2041. Based on an extrapolated linear development of incidence rates and the main alternative of population development (solid line). The colored area graphs display measured numbers over the study period. High- and low-scenario interval (dashed lines) displays the uncertainty in the population projection.

Extrapolating adjusted incidence rates and using official population projections showed substantial increases in numbers of fractures by 2041. For all major fractures combined we estimated the numbers to go from 27,284 in 2021 to 44,780 (95% prediction interval [PI] 41,375–45,324) in 2041, an increase of 64% (PI 48–70) (Figure 3, Supplementary Table S6). Upper extremity fractures could be expected to have a steeper increase than the other groups, comprising 41% of major fractures in the elderly by 2041. Hip fractures show a modest increase, consistent with an implied assumption that incidence rates continue to drop.

We found minor differences between the low, main, and high population alternatives. Sensitivity analyses, with the adjusted incidence rates kept constant at the 2021 level, resulted in a similar total number by 2041: 45,200 (PI 43,420–46,520) projected fractures, a 66% (PI 54–78) increase from 2021 (Supplementary Table S6 and Supplementary Figure S1), with the distribution of fractures comparable to the current situation.

### Stratified analyses

#### Age and sex trends

Incidence rates generally increased with age for all fracture groups (Supplementary Figures S2 and S3). The number of fractures consistently falls with increasing age for the extremity fractures, while both the spine/pelvis and hip groups have peaks in the 85–89 age group. We find consistently higher incidence rates and numbers of fractures for women in all age groups. Overall, men have a higher proportional increase in incidence rates with age.  

## Discussion

Our aim was to calculate rates of major fractures by fracture location in elderly Norwegians over the years 2010 to 2021 and thereby estimate the volume of fractures in this population by 2041. We find that although the number of fractures increased between 2010 and 2021, we find a slight overall decrease in age- and sex-adjusted incidence rates of major fractures over the period. Hip fractures contributed greatly to the decline by a pronounced reduction in incidence rates, while other extremity fractures and spine/pelvis fractures had minor increases. Incidence rates are higher for women than for men, although numbers increased at a higher rate for men. Despite a falling incidence rate, there may be a 64% increase in fractures in this population following the expected demographic development over the coming 20 years.

### Comparing our findings with previous literature

Recent studies point to a gradually reduced incidence rate for some fractures in the elderly [10,11]. Andreasen et al. studied all adult forearm fractures from 2008 to 2019 using the same data sources as our study and found slightly decreasing incidence rates for men, but not for women [11]. This contrasts somewhat with our finding of largely unchanged incidence rates among older individuals, suggesting mechanisms behind an overall decline in fractures may not affect older individuals.

Our findings of a decline in hip fracture incidence are in line with previous studies reporting falling incidence rates. A Norwegian study on data from 1999 to 2013 found an average annual age-adjusted decline of 1.5% for women and 0.8% for men [12]. Michaëlsson et al. studied hip fractures in the Swedish patient registry, finding a 14% reduction in hip fractures from 1998 to 2019 [13]. Several reasons for these falling incidence rates have been proposed. One-fifth of the decline in hip fractures in Norway is reported to be explained by anti-osteoporotic medication, while two-thirds were attributable to other modifiable factors such as increased BMI, prevalent total hip prosthesis, increased physical activity, and declining rates of smoking [14].

Increasing rates of both spinal and pelvic fractures have been shown in previous studies, corresponding to our findings [15,16]. Notably, a Swedish study based on national patient registry data reported increased rates of pelvic fractures, particularly in the elderly [16]. The same was the case for vertebral fractures in a German study, suggesting it may partly be due to increased detection by CT and MRI imaging [15].

Net increases in fractures due to demographic changes is also concluded in other studies, particularly for hip fractures [3,17]. While most of the epidemiologic research on large cohorts is done on hip fractures, which benefit from nationwide registers in several countries, studies on other fractures are primarily done on local cohorts and consider occurrences over shorter time frames, sometimes even imputed from measured hip fracture rates [1,4]. Major secular trends of fractures in the elderly are thus less well documented. In our study, we find that incidence rates for non-hip fractures in the elderly have a slight increase over time, contrary to hip fractures, compounding the effect of population aging.

Incidence rates of major fractures are consistently higher for women than men in the elderly population. However, we found a larger decrease in incidence rates among women than men. Life expectancy in the Norwegian population increased slightly more for men than for women over the study period and is projected to continue to do so for the coming 20 years [18]. Our data show that the incidence rate for all fractures in men starts catching up with that of women from around age 80.

Awareness of preventive measures may be different for women and men. There may be less awareness regarding osteopenia and osteoporosis also being a factor in fracture risk in elderly men, as suggested by a study reporting 11% of women and only 3% of men used anti-osteoporotic medication in the first year after fracture [19].

### Limitations

Registry data may be prone to both under- and overreporting. Data quality relies on precise coding by clinicians. Systematic errors in coding practices may skew the absolute numbers and incidence rates but should not substantially affect the secular trends we present.

Overestimating fracture rates due to miscoding of follow-up can affect data quality. A recent study demonstrates a large discrepancy between a naive approach and a model-based approach for identifying incident fractures from a similar data source [13]. We implemented a 6-month wash-out period, preventing individuals entering with the same fracture diagnosis more than once within that time. This approach, similar to a study by Omsland et al., demonstrated 90% sensitivity and positive predictive values when identifying forearm fractures in the NPR [8]. We subtracted the wash-out period from time at risk, which reduces the impact of the wash-out on calculated incidence rates.

Because individuals are at a higher risk of additional fractures within the first year after a major fracture, true incident fractures might be excluded due to the wash-out [20]. In our study, less than 10% of fracture episodes were excluded, likely representing an upper bound for the actual number. Incidence rates would be less affected because time at risk was adjusted accordingly.

Our study includes only patients reported to the NPR. This could be a limitation to validity in that some patients with incident fractures may not be included in the study. A Norwegian study found that 7% of cases of adult forearm fractures were treated exclusively in primary care [21]. Other fractures may not come to medical attention at all, such as spinal compression fractures, which may go undetected in as much as 2/3 of cases [14]. Even so, our findings should approximate the true numbers receiving treatment in Norwegian hospitals.

### Strengths

A major strength of our study is the use of comprehensive registry data to estimate nationwide incidence rates for major fractures in elderly Norwegians. The NPR has a high level of completeness [6]. Hip fractures may still serve as a benchmark for validity, being well documented in the Norwegian Hip Fracture Registry (NHFR). For 2019 the Hip Fracture Registry reports 7,877 operations for primary hip fractures, while our number for incident hip fractures the same year is 8,269. The NHFR gathers information on operatively treated fractures only. The completeness of the NHFR is 94%, 88%, and 91% for hemiarthroplasty, osteosynthesis, and total hip arthroplasty respectively. The same numbers for NPR were 98%, 97%, and 95% [22]. The somewhat higher number of hip fractures in our data is thus warranted and supports that NPR data is a close approximation to the true incidence.

The long duration of our study provides statistical power and potential to avoid coincidental effects. A pronounced dip in overall incidence rates in 2020 is likely to be related to the COVID-19 pandemic and its effect on hospital admissions, and to a certain extent people’s mobility due to social restrictions. The dip in upper extremities fracture rates in 2014/15 may be due to an unusually mild winter in Norway. Colder temperatures have been shown to be associated with an increased incidence of forearm fractures in the Norwegian setting [23].

While specific to Norway, the patterns observed may inform trends in other regions facing similar demographic challenges. Several studies have commented on population aging eclipsing falling incidence rates of hip fractures across the globe [3,17,24]. Our study confirms this and adds that non-hip fractures in the elderly may have an even more profound impact on fracture care in the future. While Nordic countries have some of the highest fragility-related fracture rates, there are indications that other regions may be catching up [10]. An increased focus on these other major fractures may therefore be warranted globally.

### Conclusions

While we see declining incidence rates for hip fractures, which constitute a large proportion of fractures, other major fractures have stable or slightly increasing incidence rates in the elderly. We can expect a substantial increase in the number of fractures over the coming years due to the expected aging of the population.

*In perspective,* our findings indicate that non-hip fracture rates diverge in trends, suggesting a need for more nuanced projections and costs analyses, which may be crucial for policymakers to prioritize limited resources.

### Supplementary data

Supplementary Tables S1–S6 and Figures S1–S3 are available as Supplementary data on the article page, doi: 10.2340/17453674.2024.42634

## Supplementary Material



## Notes

JA: study concept, manuscript drafting, data analysis; AA: concept development, co-writing, statistical analysis; LGJ: concept idea and development, co-writing; SMN, GMW: concept development, co-writing.

Handling co-editors: Cecilia Rogmark and Robin Christensen

*Acta* thanks Liza N van Steenbergen and David Wennergren for help with peer review of this manuscript.
