# Supplementary material for: Falling rates but projected rising numbers of fractures in elderly Norwegians: a study of fracture rates in the Norwegian patient registry from 2010 to 2021, extrapolated to 2041
Source: Acta Orthop. 2025 Feb 24;96:182–8. doi: 10.2340/17453674.2024.42634 (PMC11851338; doi:10.2340/17453674.2024.42634)

Supplementary Table S1. Fracture selection by ICD-10

Fractures included in the study, grouped by anatomic region and their corresponding ICD-10 code

Fracture group	Fracture location	ICD-10 code
Upper extremity	Clavicle	S42.0
	Scapula	S42.1
	Proximal humerus	S42.2
	Humerus shaft	S42.3
	Distal humerus	S42.4
	Proximal ulna	S52.0
	Proximal radius	S52.1
	Forearm shaft	S52.2, S52.3, S52.4
	Distal forearm	S52.5, S52.6
Spine and pelvis	Cervical spine	S12.0-9
	Thoracolumbar spine	S22.0, S22.1, S32.0
	Pelvis	S32.1-5, S32.7
Lower extremity	Femur excl. hip	S72.3, S72.4, S72.7
	Leg	S82.0-4, S82.7
	Ankle	S82.5, S82.6, S82.8
Hip	Proximal femur	S72.0-2

Supplementary Table S2. All major fractures 2010–2021

Numbers (n) of fractures, person-years at risk (PY) and incidence rates (IR) for all major fractures combined in men and women respectively, as well as proportion of fractures occurring in women. Total change in numbers and incidence rates from first to the last year of the study.

Year	Men			Women			Total		
	n	PY *1,000 [a]	IR [b]	n	PY *1,000 [a]	IR [b]	n	PY * 1,000 [a]	IR [b]
**2010**	5,524	333	1,657	17,057	425	4,015	22,581	758	2,979
**2011**	5,901	348	1,697	17,475	436	4,008	233,76	784	2,982
**2012**	5,805	362	1,605	17,340	447	3,878	23,145	809	2,861
**2013**	5,923	373	1,587	17,844	455	3,924	23,767	828	2,870
**2014**	5,696	386	1,477	17,142	464	3,693	22,838	850	2,687
**2015**	6,212	397	1,563	17,413	473	3,682	23,625	870	2,716
**2016**	6,546	410	1,597	1,8865	483	3,907	25,411	893	2,846
**2017**	7,110	421	1,689	19,175	491	3,906	26,285	912	2,882
**2018**	7,075	433	1,634	19,509	501	3,895	26,584	934	2,846
**2019**	7,336	446	1,646	19,502	512	3,812	26,838	958	2,801
**2020**	6,848	460	1,489	18,360	524	3,502	252,08	984	2,562
**2021**	7,579	472	1,606	20,017	534	3,746	27,596	1,006	2743
Δ	2,055	139	–51	2960	109	–269	5015	248	–236
Δ %	37%	42%	–3%	17%	26%	–7%	22 %	33%	–8%

[a] person-years at risk, [b] incidence rate per 100,000 person-years at risk

Supplementary Table S3. Summarizes the fractures by anatomic group: Numbers (n) and the calculated incidence rates (IR) for men, women and the combined total for each year of the study

		Men		Women		Total	
**Fracture group**	**Year**	**n**	**IR[a]**	**n**	**IR[a]**	**n**	**IR[a]**
**Upper extremity**	2010	1,510	453	6,746	1,587	8,256	1,089
	2011	1,626	467	6,941	1,591	8,567	1,093
	2012	1,601	442	6,887	1,540	8,488	1,049
	2013	1,637	438	7,185	1,580	8,822	1,065
	2014	1,531	397	6,658	1,434	8,189	963
	2015	1,674	421	6,956	1,470	8,630	991
	2016	1,875	457	7,840	1,623	9,715	1,088
	2017	2,100	499	8,210	1,672	10,310	1,130
	2018	2,065	477	8,495	1,696	10,560	1,131
	2019	2,154	483	8,456	1,652	10,610	1,108
	2020	1,979	430	7,860	1,499	9,839	1,000
	2021	2,261	479	8,730	1,633	10,991	1,092
**Spine and pelvis**	2010	828	248	2,026	477	2,854	376
	2011	861	248	1,953	448	2,814	359
	2012	941	260	1,997	446	2,938	363
	2013	961	257	2,057	452	3,018	364
	2014	910	236	2,084	449	2,994	352
	2015	996	251	2,149	454	3,145	361
	2016	1,090	266	2,275	471	3,365	377
	2017	1,211	288	2,343	477	3,554	390
	2018	1,191	275	2,245	448	3,436	368
	2019	1,239	278	2,457	480	3,696	386
	2020	1,277	278	2,293	437	3,570	363
	2021	1,397	296	2,568	480	3,965	394
**Lower extremity excluding hip**	2010	853	256	2,454	577	3,307	436
	2011	929	267	2,624	602	3,553	453
	2012	885	245	2,540	568	3,425	423
	2013	912	244	2,634	579	3,546	428
	2014	898	233	2,563	552	3,461	407
	2015	1,029	259	2,639	558	3,668	421
	2016	1,040	254	2,899	600	3,939	441
	2017	1,153	274	3,009	613	4,162	456
	2018	1,196	276	3,108	620	4,304	461
	2019	1,236	277	3,022	591	4,258	445
	2020	1,080	235	2,904	554	3,984	405
	2021	1,237	262	3,209	600	4,446	442
**Hip**	2010	2,333	701	5,831	1,379	8,164	1,081
	2011	2,485	717	5,957	1,374	8,442	1,082
	2012	2,378	659	5,916	1,331	8,294	1,030
	2013	2,413	648	5,968	1,320	8,381	1,017
	2014	2,357	613	5,837	1,264	8,194	968
	2015	2,513	634	5,669	1,205	8,182	944
	2016	2,541	621	5,851	1,218	8,392	944
	2017	2,646	630	5,613	1,149	8,259	909
	2018	2,623	607	5,661	1,136	8,284	890
	2019	2,707	609	5,567	1,093	8,274	867
	2020	2,512	547	5,303	1,016	7,815	797
	2021	2,684	570	5,510	1,035	8,194	817

[a] Incidence rate per 100,000 person-years at risk

Supplementary Table S4. Component fracture location

Summarizes the sixteen component fractures. Numbers (n) and the calculated incidence rate (IR)s for men, women and the combined total for each year of the study.

		Men		Women		Total	
**Fracture location**	**Year**	**n**	**IR**	**n**	**IR**	**n**	**IR**
**Clavicle**	2010	210	63	315	74	525	69
	2011	201	58	316	72	517	66
	2012	183	51	289	65	472	58
	2013	199	53	308	68	507	61
	2014	193	50	286	62	479	56
	2015	205	52	336	71	541	62
	2016	267	65	398	82	665	74
	2017	266	63	388	79	654	72
	2018	295	68	373	74	668	71
	2019	269	60	396	77	665	69
	2020	271	59	384	73	655	67
	2021	311	66	400	75	711	71
**Scapula**	2010	44	13	49	12	93	12
	2011	42	12	47	11	89	11
	2012	37	10	68	15	105	13
	2013	51	14	50	11	101	12
	2014	46	12	67	14	113	13
	2015	57	14	63	13	120	14
	2016	50	12	74	15	124	14
	2017	62	15	81	16	143	16
	2018	56	13	86	17	142	15
	2019	62	14	92	18	154	16
	2020	86	19	81	15	167	17
	2021	81	17	92	17	173	17
**Proximal humerus**	2010	419	126	1,809	426	2,228	294
	2011	450	129	1,967	452	2,417	309
	2012	457	126	1,896	425	2,353	291
	2013	473	127	2,056	453	2,529	306
	2014	436	113	1,895	409	2,331	274
	2015	490	123	1,971	417	2,461	283
	2016	500	122	2,167	449	2,667	299
	2017	582	138	2,334	476	2,916	320
	2018	580	134	2,300	460	2,880	309
	2019	628	141	2,385	467	3,013	315
	2020	505	110	2,135	408	2,640	268
	2021	635	135	2,432	456	3,067	305

**Humerus shaft**	2010	58	17	189	44	247	33
	2011	65	19	199	46	264	34
	2012	72	20	176	39	248	31
	2013	57	15	186	41	243	29
	2014	59	15	180	39	239	28
	2015	64	16	184	39	248	28
	2016	77	19	189	39	266	30
	2017	85	20	179	36	264	29
	2018	77	18	184	37	261	28
	2019	70	16	217	42	287	30
	2020	69	15	181	34	250	25
	2021	79	17	216	40	295	29
**Distal humerus**	2010	42	13	161	38	203	27
	2011	59	17	161	37	220	28
	2012	45	12	175	39	220	27
	2013	46	12	185	41	231	28
	2014	48	12	170	37	218	26
	2015	50	13	148	31	198	23
	2016	65	16	185	38	250	28
	2017	60	14	174	35	234	26
	2018	60	14	187	37	247	26
	2019	71	16	186	36	257	27
	2020	73	16	200	38	273	28
	2021	75	16	213	40	288	29
**Proximal ulna**	2010	65	19	212	50	277	37
	2011	63	18	235	54	298	38
	2012	54	15	265	59	319	39
	2013	58	16	222	49	280	34
	2014	56	15	196	42	252	30
	2015	71	18	219	46	290	33
	2016	69	17	281	58	350	39
	2017	93	22	233	47	326	36
	2018	80	18	246	49	326	35
	2019	84	19	239	47	323	34
	2020	69	15	230	44	299	30
	2021	98	21	266	50	364	36
**Proximal radius**	2010	34	10	159	37	193	25
	2011	54	16	155	36	209	27
	2012	45	12	185	41	230	28
	2013	45	12	189	42	234	28
	2014	56	15	164	35	220	26
	2015	51	13	183	39	234	27
	2016	69	17	215	44	284	32
	2017	83	20	223	45	306	34
	2018	79	18	246	49	325	35
	2019	75	17	241	47	316	33
	2020	67	15	257	49	324	33
	2021	70	15	213	40	283	28

**Forearm shaft**	2010	43	13	225	53	268	35
	2011	52	15	204	47	256	33
	2012	58	16	221	49	279	34
	2013	60	16	235	52	295	36
	2014	53	14	240	52	293	34
	2015	59	15	218	46	277	32
	2016	59	14	240	50	299	33
	2017	67	16	254	52	321	35
	2018	62	14	230	46	292	31
	2019	71	16	225	44	296	31
	2020	71	15	241	46	312	32
	2021	75	16	254	47	329	33
**Distal forearm**	2010	595	179	3,627	856	4,222	558
	2011	640	184	3,657	842	4,297	550
	2012	650	180	3,612	811	4,262	528
	2013	648	174	3,754	829	4,402	533
	2014	584	151	3,460	748	4,044	477
	2015	627	158	3,634	771	4,261	491
	2016	719	176	4,091	850	4,810	540
	2017	802	191	4,344	888	5,146	566
	2018	776	179	4,643	931	5,419	582
	2019	824	185	4,475	878	5,299	555
	2020	768	167	4,151	795	4,919	501
	2021	837	177	4,644	873	5,481	546
**Cervical spine**	2010	87	26	80	19	167	22
	2011	116	33	96	22	212	27
	2012	145	40	88	20	233	29
	2013	131	35	107	24	238	29
	2014	140	36	130	28	270	32
	2015	139	35	115	24	254	29
	2016	162	40	141	29	303	34
	2017	160	38	131	27	291	32
	2018	185	43	131	26	316	34
	2019	184	41	145	28	329	34
	2020	221	48	119	23	340	35
	2021	200	42	136	25	336	33
**Thoracolumbar spine**	2010	455	137	980	231	1,435	189
	2011	429	123	842	193	1,271	162
	2012	445	123	843	188	1,288	159
	2013	445	119	914	201	1,359	164
	2014	450	117	871	188	1,321	155
	2015	484	122	894	189	1,378	158
	2016	499	122	893	185	1,392	156
	2017	566	135	906	184	1,472	161
	2018	504	116	901	180	1,405	150
	2019	542	122	983	192	1,525	159
	2020	575	125	942	180	1,517	154
	2021	653	138	1,078	202	1,731	172

**Pelvis**	2010	286	86	966	227	1,252	165
	2011	316	91	1,015	233	1,331	170
	2012	351	97	1,066	238	1,417	175
	2013	385	103	1,036	228	1,421	172
	2014	320	83	1,083	233	1,403	165
	2015	373	94	1,140	241	1,513	174
	2016	429	105	1,241	257	1,670	187
	2017	485	115	1,306	266	1,791	196
	2018	502	116	1,213	242	1,715	184
	2019	513	115	1,329	260	1,842	192
	2020	481	105	1,232	235	1,713	174
	2021	544	115	1,354	253	1,898	189
**Femur excluding hip**	2010	134	40	541	127	675	89
	2011	129	37	581	133	710	91
	2012	121	33	556	124	677	84
	2013	122	33	568	125	690	83
	2014	128	33	520	112	648	76
	2015	129	32	480	101	609	70
	2016	113	28	556	115	669	75
	2017	149	35	535	109	684	75
	2018	152	35	532	106	684	73
	2019	147	33	500	98	647	68
	2020	117	25	459	87	576	58
	2021	147	31	513	96	660	66
**Lower leg**	2010	324	97	883	208	1,207	159
	2011	382	110	957	219	1,339	171
	2012	367	101	969	217	1,336	165
	2013	397	106	970	213	1,367	165
	2014	399	103	989	213	1,388	163
	2015	448	113	1,062	225	1,510	174
	2016	421	103	1,119	232	1,540	173
	2017	479	114	1,186	242	1,665	183
	2018	465	107	1,177	235	1,642	176
	2019	555	125	1,191	233	1,746	182
	2020	469	102	1,173	224	1,642	167
	2021	528	112	1,243	233	1,771	176
**Ankle**	2010	395	119	1,030	242	1,425	188
	2011	418	120	1,086	249	1,504	192
	2012	397	110	1,015	227	1,412	175
	2013	393	105	1,096	241	1,489	180
	2014	371	96	1,054	227	1,425	168
	2015	452	114	1,097	232	1,549	178
	2016	506	123	1,224	254	1,730	194
	2017	525	125	1,288	262	1,813	199
	2018	579	134	1,399	279	1,978	212
	2019	534	120	1,331	260	1,865	195
	2020	494	107	1,272	243	1,766	179
	2021	562	119	1,453	272	2,015	200

**Hip**	2010	2,333	701	5,831	1,379	8,164	1,081
	2011	2,485	717	5,957	1,374	8,442	1,082
	2012	2,378	659	5,916	1,331	8,294	1,030
	2013	2,413	648	5,968	1,320	8,381	1,017
	2014	2,357	613	5,837	1,264	8,194	968
	2015	2,513	634	5,669	1,205	8,182	944
	2016	2,541	621	5,851	1,218	8,392	944
	2017	2,646	630	5,613	1,149	8,259	909
	2018	2,623	607	5,661	1,136	8,284	890
	2019	2,707	609	5,567	1,093	8,274	867
	2020	2,512	547	5,303	1,016	7,815	797
	2021	2,684	570	5,510	1,035	8,194	817

[a] incidence rate per 100,000 person-years at risk

Supplementary Table S5. Estimated percentage annual change in numbers and adjusted annual change in incidence rate for the 16 component fracture locations.

Fracture location	Estimated annual change [%] in number of fractures (CI)	Estimated adjusted annual change [%] in incidence rate (CI)
Clavicle	3.7 (3.0 to 4.4)	1.4 (0.7 to 2.1)
Scapula	6.4 (4.8 to 7.9)	3.9 (2.4 to 5.5)
Proximal humerus	2.6 (2.3 to 3.0)	0.6 (0.2 to 0.9)
Humerus shaft	1.2 (0.1 to 2.2)	–0.9 (–1.9 to 0.1)
Distal humerus	2.8 (1.7 to 3.9)	0.8 (–0.3 to 1.8)
Proximal ulna	1.7 (0.8 to 2.7)	–0.3 (–1.3 to 0.6)
Proximal radius	4.6 (3.6 to 5.7)	2.3 (1.2 to 3.3)
Forearm shaft	1.7 (0.7 to 2.6)	–0.5 (–1.4 to 0.5)
Distal forearm	2.7 (2.4 to 2.9)	0.5 (0.3 to 0.8)
Cervical spine	5.5 (4.5 to 6.6)	3.0 (2.0 to 4.0)
Thoracolumbar spine	1.9 (1.5 to 2.4)	–0.1 (–0.5 to 0.3)
Pelvis	3.7 (3.2 to 4.1)	2.0 (1.6 to 2.4)
Femur excluding hip	–0.8 (–1.4 to –0.2)	–2.4 (–3.0 to –1.8)
Lower leg	3.3 (2.9 to 3.7)	1.0 (0.6 to 1.4)
Ankle	3.4 (3.0 to 3.8)	1.0 (0.6 to 1.4)
Hip	–0.2 (–0.4 to –0.1)	–1.9 (–2.0 to –1.7)

Supplementary Table S6. Projected number of fractures from 2021 to 2041.

Calculated using incidence rates adjusted for age, age with a quadratic term and sex, using Poisson regression, with time at risk included as an offset-parameter. 95% prediction intervals (PI) calculated by bootstrapping. We present two scenarios based on the main alternative for population development provided by Statistics Norway; Left: A linear projection of incidences rates, which assumes that incidence rates continue to develop as during the years 2010-2021. Right: Assuming unchanged incidence rates from 2021 and onward.

PI: Prediction interval

Fracture group	Year	Projection model			
		Linear IR	(PI)	Fixed IR	(PI)
**All major fractures**	2021	27,284	(25,658 to 28,542)	27,284	(25,742 to 28,790)
	2041	44,780	(41,375 to 45,324)	45,228	(43,420 to 46,520)
	Δ 2021–2041	17,496	(13,439 to 18,077)	17,944	(15,332 to 20,171)
	Δ %	64.1	(47.6 to 69.9)	65,8	(54.0 to 78.0)
**Upper extremity**	2021	10,803	(9,955 to 11,609)	10,803	(9,630 to 11,407)
	2041	18,514	(17,630 to 19,348)	16,506	(15,310 to 17,147)
	Δ 2021–2041	7,711	(6,386 to 9,049)	5,704	(4,299 to 7,105)
	Δ %	71.4	(55.9 to 90.1)	52.8	(38.4 to 72.8)
**Spine and pelvis**	2021	3859	(3,639 to 4,114)	3859	(3,385 to 3,977)
	2041	8575	(8,322 to 8,864)	6817	(,6326 to 7,006)
	Δ 2021–2041	4716	(4,355 to 5,089)	2958	(2,475 to ,3464)
	Δ %	122.2	(107 to 138)	76.6	(62.6 to 100)
**Lower extremity excluding hip**	2021	4370	(4,036 to 4720)	4370	(3,983 to 4,658)
	2041	7267	(6,919 to 7635)	6694	(6,293 to 7,004)
	Δ 2021–2041	2897	(2,404 to 3,404)	2323	(1,802 to 2,845)
	Δ %	66.3	(51.9 to 83.4)	53.2	(39.4 to 70.2)
**Hip**	2021	8252	(7,750 to 8,584)	8252	(7,959 to 9,757)
	2041	10 424	(9,918 to 10,769)	15211	(14,889 to 16,763)
	Δ 2021–2041	2172	(1,575 to 2,784)	6959	(5,482 to 8,408)
	Δ %	26.3	(18.6 to 35.8)	84.3	(57.4 to 104)

Supplementary Figure S1. Projected number of fractures in 2 extrapolation scenarios.

Projections calculated using estimated incidence rates adjusted for age, age with a quadratic term and sex, using a Poisson regression model, with time at risk included as an offset-parameter. We present 2 scenarios based on the main alternative for population development provided by Statistics Norway: A linear projection of incidences rates, which assumes that incidence rates continue to develop as during the years 2010–2021 (solid line). Assuming unchanged incidence rates from 2021 and onward (dashed line). Actual numbers from 2010 to 2021 are shown as dots.

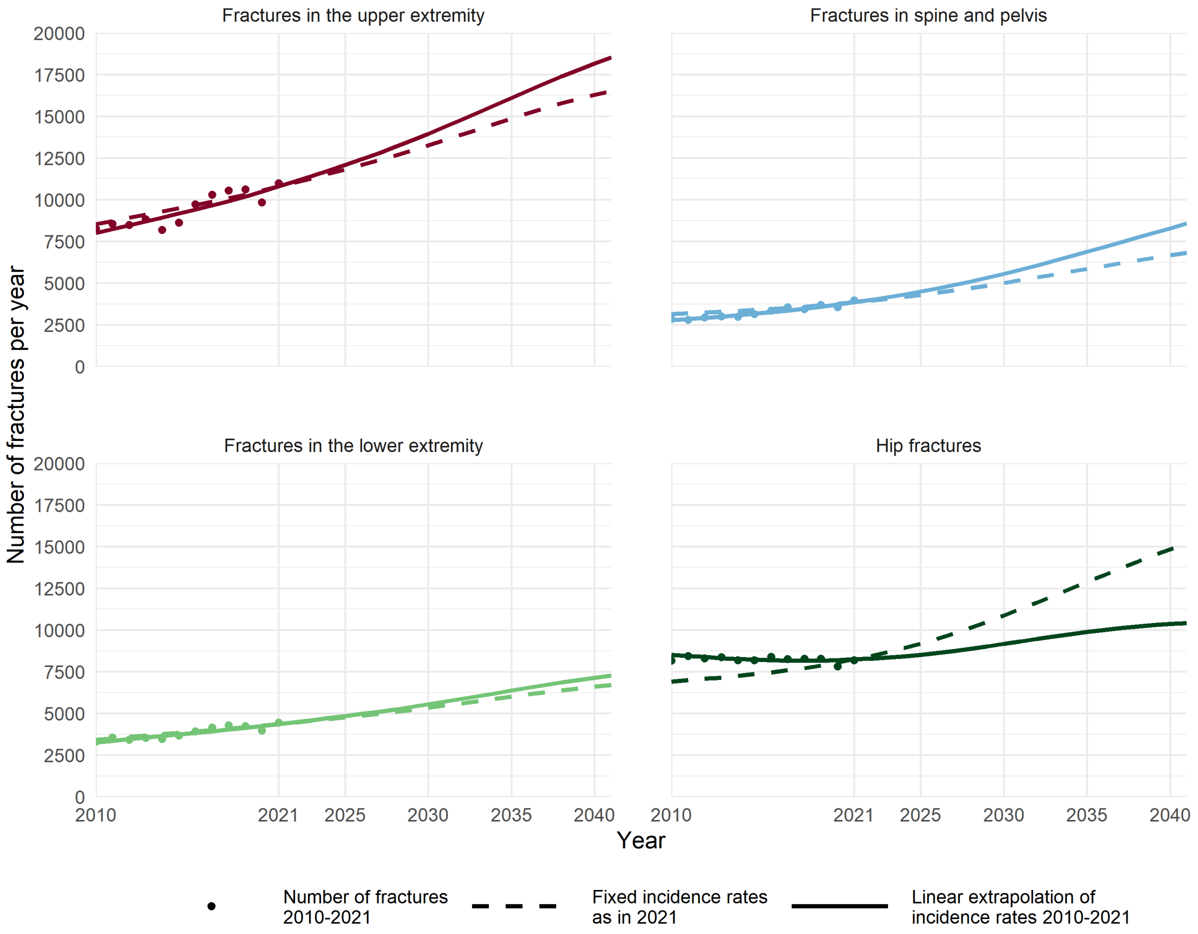

Supplementary Figure S2. Mean number of fractures per year in men and women from 2010 to 2021, total and by anatomic fracture group, in 5-year age brackets

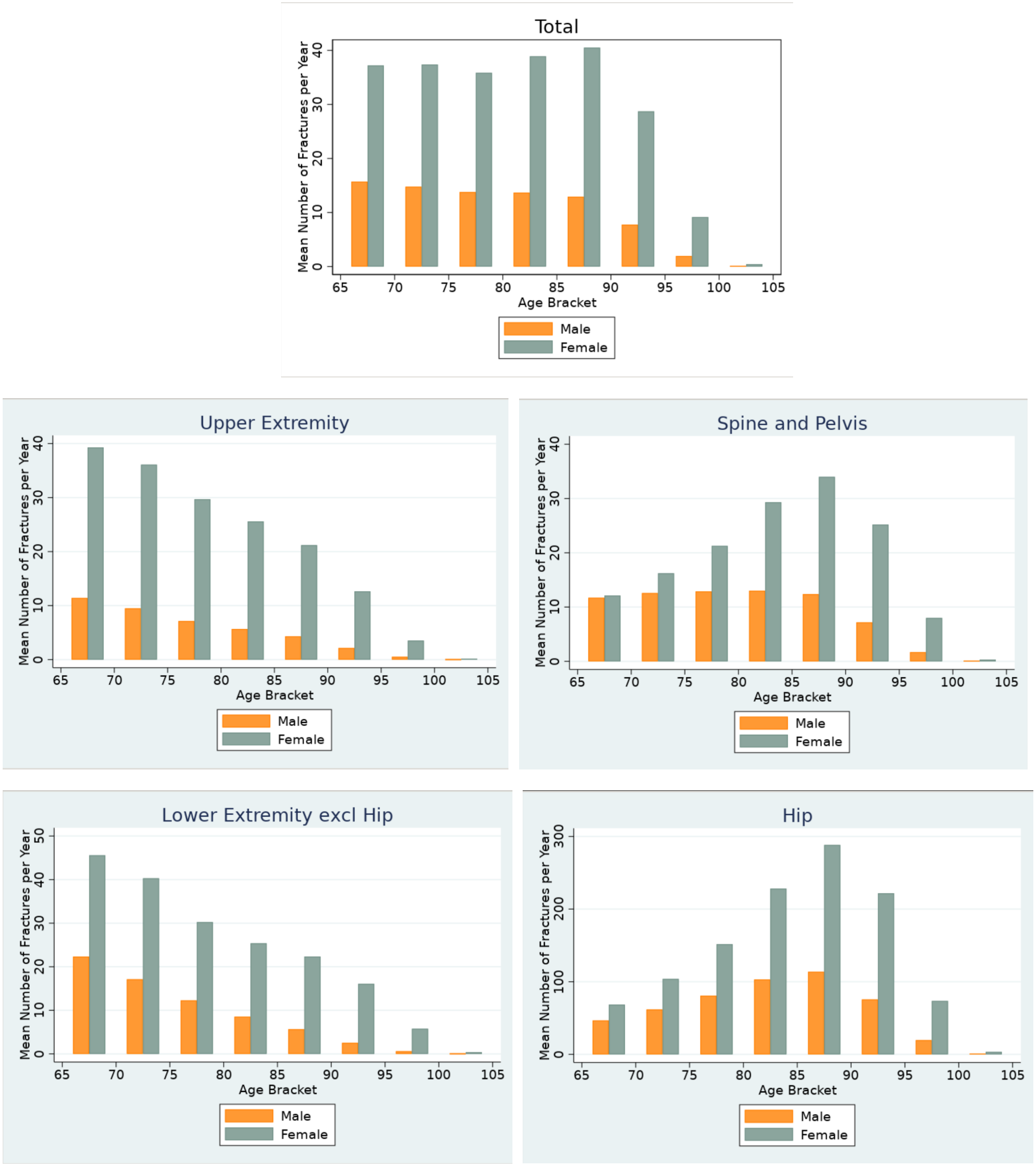

Supplementary Figure S3. Mean incidence rates of fractures per year in men and women from 2010 to 2021, by anatomic fracture group, in 5-year age brackets

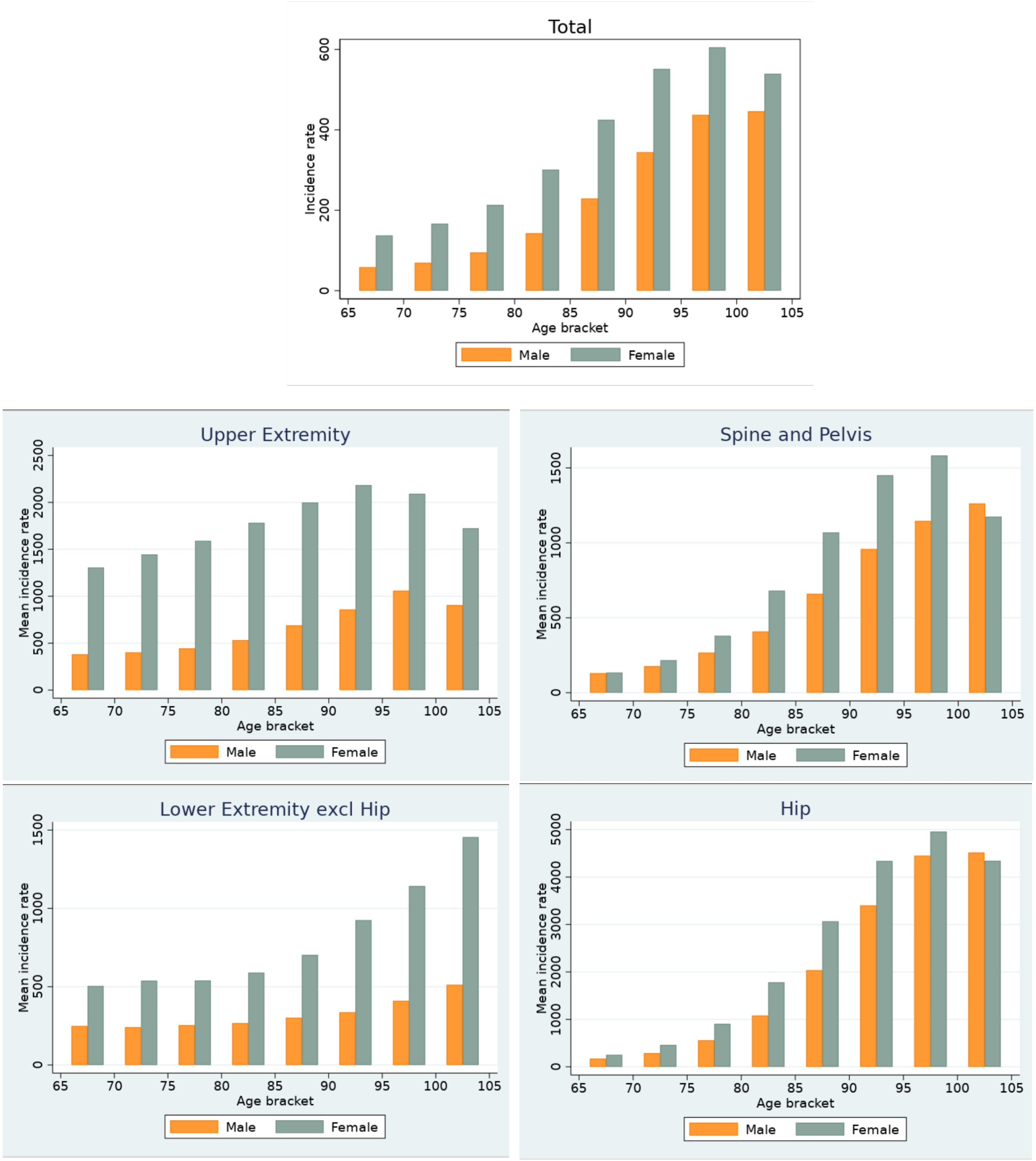

Supplement: Supplementary file 1 [file ActaO-96-42634-s1.pdf]
